# Comparison of the effects of bimatoprost and a fixed combination of latanoprost and timolol on 24-hour blood and ocular perfusion pressures: the results of a randomized trial

**DOI:** 10.1186/1471-2415-15-7

**Published:** 2015-01-22

**Authors:** Luca Rossetti, Matteo Sacchi, Costas H Karabatsas, Fotis Topouzis, Michele Vetrugno, Marco Centofanti, Andreas Boehm, Christian Vorwerk, David Goldblum, Paolo Fogagnolo

**Affiliations:** Clinica Oculistica, Dipartimento di Medicina, Chirurgia e Odontoiatria, Università di Milano, Ospedale San Paolo, Via di Rudinì 8, 20142 Milan, Italy; Department of Ophthalmology, University of Thessaly School of Medicine, Larissa, Greece; Department of Ophthalmology, School of Medicine, Aristotle University of Thessaloniki, American Hellenic Educational Progressive Association (AHEPA) Hospital, Thessaloniki, Greece; Anthea Hospital, GVM Care and Research, Bari, Italy; DSCMT Università di Roma ‘Tor Vergata’; IRCCS Fondazione G.B. Bietti, Rome, Italy; University Hospital Carl Gustav Carus Dresden, Technical University Dresden, Dresden, Germany; Augenklinik der Otto von Guericke Universität Magdeburg, Magdeburg, Germany; Department of Ophthalmology, University Hospital Basel, University Basel, Basel, Switzerland

## Abstract

**Background:**

To compare the effect of bimatoprost and the fixed combination latanoprost-timolol (LTFC) on 24-hour systolic (SBP) and diastolic (DBP) blood pressure and on 24-hour ocular perfusion pressure (OPP).

**Methods:**

200 patients with glaucoma or ocular hypertension, controlled on the unfixed combination of latanoprost and timolol or eligible for dual therapy being not being fully controlled on monotherapy were enrolled in a randomized, double-masked, placebo-controlled, multicentre clinical trial. They were randomized to LTFC (8 a.m.) or bimatoprost (8 p.m.) and received 24-hour IOP curve at baseline, 6 and 12 weeks (supine and sitting position IOPs were recorded at 8 p.m., midnight, 5 a.m., 8a.m., noon and 4 p.m.). Holter 24-hour blood pressure curve was obtained between weeks 2 and 12. SBP, DBP, OPP were calculated and compared with ANOVA. Rates of diastolic OPP (DPP) ≤50, ≤40, ≤30 mmHg in the 2 groups were calculated and compared using Fisher’s test.

**Results:**

Mean baseline SBP and DBP were 136.5 ± 18.3 vs 134.2 ± 20.1 mmHg (p = 0.1) and 79.1 ± 10.2 vs 78.2 ± 10.1 mmHg (p = 0.4) in the bimatoprost and LTFC groups respectively. Holter SBP was significantly higher for bimatoprost (135.1 mmHg vs 128.1 mmHg, p = 0.04), while no statistically significant difference in DBP was found. DPP was similar in the 2 groups, and proportions of patients with at least one value of the 24-hour curve ≤50, ≤40, ≤30 mmHg were 94%, 86%, 41% respectively.

**Conclusions:**

Bimatoprost and LTFC had similar DBPs and OPPs; SBP was significantly lower with LTFC. In this study, the percentage of “dippers” was considerably higher than the one described in previous studies on the role of perfusion pressure in glaucoma.

**Trial registration:**

NCT02154217, May 21, 2014.

## Background

Primary open angle glaucoma (POAG) is a disease whose pathogenesis is not completely clear [1]. There is evidence that elevated intraocular pressure (IOP) is a strong risk factor for both occurrence and progression of glaucoma [2, 3] and also that IOP reduction can be highly protective from worsening of the disease [4]. However, IOP alone can hardly explain all of the pathogenesis of glaucoma and the importance of vascular factors, in particular of perfusion pressure (PP), has been ascertained in a number of epidemiologic studies and clinical trials as well [5–8]. Low values of diastolic perfusion pressure (DPP) have been reported to be a major risk factor for the incidence of glaucoma [9, 10], and a decreased DPP was found to be a significant predictor for glaucoma progression [11–13]. Yet, the “independent” role of PP in the pathogenesis of the disease is debated [14]; a further issue is the way PP is calculated and assessed in multivariate analyses together with IOP [15]. Evaluation of both IOP and PP is often a critical point in the context of an epidemiologic study and in a clinical trial as well. Both parameters can, in fact, considerably vary during the 24-hour and can be susceptible to a number of other variables (time point of the day or night, concomitant use of systemic or local drugs, emotional stress during the measurement, etc.). Probably the best way to evaluate IOP rhythm is by means of 24-hour measurements [16–20], while the 24-hour Holter recording is the most appropriate procedure to get an estimate of the systemic blood pressure during a 24-hour period.

The effect of beta-blockers and prostaglandin analogues on IOP reduction is well known [21], whereas these drugs’ effect on PP has been studied only more recently [22, 23]. A European multicenter randomized clinical trial was conducted with the aim of comparing the effectiveness and safety of bimatoprost and of the LTFC in lowering IOP in patients with POAG or OH when switched from a non-fixed combination of latanoprost and timolol, and the findings have been published [24]. The objective of this paper was to investigate the effect of these drugs on PP, assess the number of patients “at risk” due to low DPP despite apparently good IOP control and to calculate the proportion of time in a day when patients were, on average, below PP values that we conventionally consider as “potentially safe”.

## Methods

The study was carried out at 7 European University Eye Clinics after the approval of the Ethics Committee of the Universities of Milan, Rome, Bari (Italy), Larissa, Thessaloniki (Greece), Dresden, Magdeburg (Germany), Basel (Switzerland). All patients signed a written informed consent form. The patients and the methods of this trial have been described in a previous paper [24]; in this paper, we described in detail the methods for evaluation of IOP and blood pressure and their analyses. Patients’ inclusion criteria were the following:

*Inclusion criteria*:

- POAG/PEX or OH patients aged 18 years or more
- patients controlled (IOP <21 mmHg) on the unfixed combination of latanoprost and timolol for at least 3 months prior to the baseline visit.
- patients on monotherapy either with latanoprost or timolol eligible for dual therapy (IOP >21 mmHg, or target IOP not reached).

*Exclusion criteria*:

- contraindications to beta-blockers
- closed/barely open anterior chamber angles or history of acute angle closure.
- ocular surgery or argon laser trabeculoplasty within the last 3 months.
- ocular inflammation/infection occurring within 3 months prior to pre-trial visit.
- neovascular glaucomas.
- hypersensitivity to benzalkonium chloride or to any other component of the trial drug solutions.
- other abnormal ocular condition or symptom preventing the patient from entering the trial.
- patients on either bimatoprost or the LTFC
- patients who had undergone refractive surgery
- inability to adhere to treatment/visit plan.
- participation in any other clinical trial (i.e., requiring informed consent) within one month prior to pre-study visit.
- pregnancy, nursing, or, if applicable, not using adequate contraception.
- any drug known to affect IOP.

### Study design

This was a 12-week, multicentre, randomized, double-masked study. The trial was conducted in accordance with the ethical principles that have their origins in the Declaration of Helsinki and its amendment of October 2000 (Edinburgh, Scotland), the European Guidelines on Good Clinical Practice (GCP) and the International Conference on Harmonisation (ICH) Guidelines. The trial included five visits: 1) patients were checked for eligibility at the pre-trial screening visit; if potentially eligible, only patients on monotherapy started wash-in medications (unfixed combination of latanoprost and timolol) after the screening visit; 2) baseline visit after at least 6 weeks of wash-in; 3) week 2 visit (safety visit including biomicroscopy, single IOP measurement and evaluation of adverse events); 4) week 6 visit; 5) week 12 visit. At the end of the study (3 weeks after week 12 visit) a safety visit was optional.

The baseline visit and the week 6 and 12 visits required hospitalization and patients were evaluated for 24 hours. Patients were hospitalized at 7 pm to undergo a 24-hour IOP assessment. Supine and sitting position IOP was measured at 8 pm, midnight, 5 am, 8 am, noon, and 4 pm; 15 minutes around the time point were allowed. At 8 pm and at 8 am, the study drops were administered by the study personnel. Both 8 a.m. and 8 p.m. tonometric recordings were performed before study drugs were administered. Supine IOP was measured using the handheld Perkins tonometer with the patient resting on a bed for at least 10–15 minutes. Sitting IOP was measured with the Goldmann applanation tonometer. At each time point IOP was measured twice, and then averaged. If the 2 recordings were not within 2 mmHg a third measurement was taken and the average calculated. All assessments were performed by the same well-trained evaluators different from the study personnel administrating the drugs.

On an outpatient basis, the patients underwent a Holter 24-hour blood pressure recording not earlier than 2 weeks after having started the trial medications.

### Study treatments

Patients on monotherapy (either latanoprost or timolol) underwent a wash-in phase lasting 6 weeks. Wash-in treatment consisted in timolol (Timolol XE morning administration) and latanoprost (Xalatan evening administration). After baseline 24-hour evaluation, each patient was allocated to one of the two treatment groups according to a centralized computer-generated randomisation code list prepared and kept at each institute by an independent administrator: 1) LTFC (Xalacom) at 8 am + placebo at 8 pm; 2) placebo at 8 am + bimatoprost (Lumigan) at 8 pm. Identical bottles were used for masking purposes.

### Definitions

The nocturnal period was from 11 pm to 7 am. Perfusion pressure was calculated as blood pressure (systolic, diastolic) minus IOP (taken from the habitual body position readings). During the Holter acquisition, blood pressure was measured every 15 minutes as opposite to IOP, which was measured every 4 hours. For the aims of the study, it was important to consider values which were less affected as possible by disturbing factors (such as sudden awakening, change in body position, exposure to light etc.).

In order to use all available BP readings (ideally 96 per patient), we arbitrarily calculated perfusion pressure by subtracting the closest IOP reading to BP. For sake of clarity, considering the period from 10:00 pm to 9:59 am, perfusion pressures for BP collected between 10:00 pm and 1:59 am were calculated using the midnight IOP measurement; from 2:00 am to 5:59, the 4 am IOP reading was subtracted to the singles BP readings, whereas from 6:00 to to 9:59 am, the 8 am IOP reading was used.

### Statistical analysis

Comparisons between treatments and within the 2 treatment groups with baseline were carried out with ANOVA. The following parameters were compared: a. IOP at baseline, at week 6 and week 12; b. IOP difference between baseline and week 12; c. 24-hour diastolic blood pressure (DBP), 24-hour systolic blood pressure (SBP) at the time of Holter acquisition; 24-hour DPP, 24-hour SPP at the time of Holter acquisition; nocturnal DPP, nocturnal SPP at the time of Holter acquisition.

The study population (as a whole and for subgroups) was also stratified based on the mean nocturnal DPP (using cut-off values of ≤50, ≤45, ≤40, ≤35, ≤30 mmHg). For each cut-off value, mean nocturnal DPP were calculated and compared. Rates of DPP ≤50, ≤45, ≤40, ≤35, ≤30 mmHg in the 2 groups were calculated and compared using Fisher’s test. In addition, the proportion of time in the day when DPP was below a given cut-off value was calculated.

The details of sample size calculation are reported in another paper [24] and were based on the 24-hour mean change in IOP. In the estimates below, the significance level was set to 5% and the power to 80%. The numbers of 81 patients per group (1-sided test) and 102 patients per group (2-sided test) were calculated for a delta of 1.5 mmHg and a standard deviation of 3.8 mmHg. P-values were adjusted for multiple comparisons (Bonferroni corrections).

## Results

A total of 200 patients were enrolled in the trial. All patients completed the study. A protocol violation occurred for two patients: one was treated for a bacterial conjunctivitis and another started a therapy with a systemic beta-blocker during the study. All data presented in the paper refer to the intention-to-treat analysis. Per-protocol analysis showed very similar results. Patients’ main characteristics are shown in Table 1. The majority of patients had initial to moderate glaucoma and about 20% of cases had OH. Treatment for systemic hypertension was common and 40% of the whole sample was taking an antihypertensive drug.

**Table 1:** **Patients’ main characteristics**

	Bimatoprost	LTFC
Total	101	99
Age, mean (sd)	64.7 (11.5)	67.8 (10.8)
Male (%)	55 (54.5)	53 (53.5)
POAG/PEX (%)	78 (77)	80 (81)
OH (%)	23 (23)	19 (19)
MD, mean (sd)	-4.2 (2.3 dB)	-4.6 (2.7 dB)
CCT, mean (sd)	543 (38 μm)	535 (39 μm)
Systemic Drugs		
Antihypertensives (%)	42 (41)	40 (40)
Antidepressants (%)	6 (6)	8 (8)
Hypoglycemics oral (%)	11 (11)	13 (13)
Cholesterol-lowering drugs (%)	13 (13)	15 (15)
Antiarrhythmics (%)	5 (5)	4 (4)

LTFC: latanoprost/timolol fixed combination; sd: standard deviation; POAG: primary open-angle glaucoma; PEX pseudoexfoliative glaucoma; OH: ocular hypertension; MD: mean deviation; CCT: central corneal thickness.

Results about IOP and PP are reported in Table 2. Very similar IOP levels at baseline and at 12 weeks were found in the 2 groups and no statistically significant difference was shown at any comparison. Baseline (i.e. under the unfixed combination of latanoprost and timolol) mean IOP was 16.3 mmHg (3.3) vs 15.5 mmHg (2.9) in bimatoprost and LTFC groups respectively and similar figures were shown when nocturnal IOPs were analyzed. Also SBP and DBP were very similar at baseline in the 2 groups and, as expected, were significantly lower during the night (p = 0.01). There was no statistically significant difference between the study drugs for all comparisons but for Holter SBP and SPP that were significantly higher in the group treated with bimatoprost (135.1 mmHg vs 128.1 mmHg and 119.0 mmHg vs 111.8 mmHg in bimatoprost and LTFC groups respectively, p = 0.04, p = 0.03).

**Table 2:** **Comparison of intraocular, systemic and perfusion pressures (mmHg) in the 2 groups**

	Bimatoprost	LTFC	p-value
Baseline IOP (sd)	16.3 (3.3)	15.5 (2.9)	0.2
Baseline nocturnal IOP (sd)	16.9 (3.6)	16.0 (3.3)	0.3
Baseline SBP (sd)	136.5 (18.3)	134.2 (20.1)	0.1
Baseline DBP (sd)	79.1 (10.2)	78.2 (10.1)	0.4
Baseline nocturnal SBP (sd)	121.0 (13.8)	122.1 (15.8)	0.3
Baseline nocturnal DBP (sd)	72.7 (7.9)	73.2 (9.5)	0.4
Baseline SPP (sd)	120.2 (15.7)	118.7 (16.8)	0.3
Baseline DPP (sd)	62.8 (6.9)	62.7 (8.2)	0.6
Baseline nocturnal SPP (sd)	104.1 (13.1)	106.1 (16.4)	0.2
Baseline nocturnal DPP (sd)	55.8 (8.0)	57.2 (12.1)	0.1
12 week IOP (sd)	16.1 (2.5)	16.3 (3.7)	0.7
12 week nocturnal IOP (sd)	16.1 (2.6)	16.1 (3.9)	0.8
Holter SBP (sd)	135.1 (16.7)	128.1 (15.3)	0.04
Holter DBP (sd)	79.5 (8.3)	78.7 (11.8)	0.4
Holter nocturnal SBP (sd)	124.8 (14.4)	120.0 (14.5)	0.08
Holter nocturnal DBP (sd)	71.7 (7.9)	70.6 (11.3)	0.2
Holter SPP (sd)	119.0 (10.8)	111.8 (15.3)	0.03
Holter DPP (sd)	63.4 (8.0)	62.4 (11.1)	0.1
Holter nocturnal SPP (sd)	108.7 (14.4)	103.9 (17.3)	0.07
Holter nocturnal DPP (sd)	55.6 (7.4)	54.5 (12.3)	0.2

LTFC: latanoprost/timolol fixed combination; IOP: intraocular pressure; sd: standard deviation; SBP: systolic blood pressure; DBP: diastolic blood pressure; SPP: systolic perfusion pressure; DPP: diastolic perfusion pressure.

**Figure 1:**
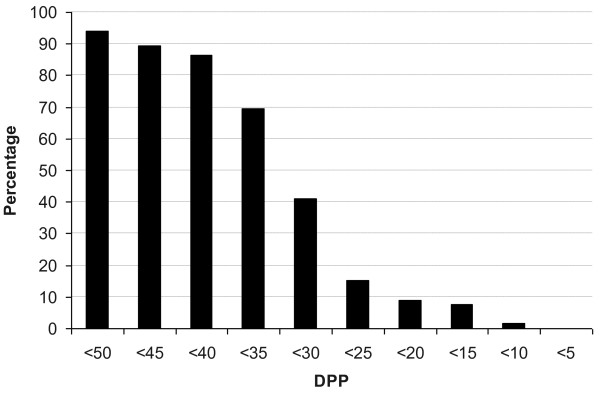
**Percentage of patients showing at least 1 DPP below the given cut-off values.**

Figure 1 is showing the proportion of patients who had at least 1 DPP reading below a given cut-off value. More than 90% of the patients had at least one DPP measurement below 50 mmHg, more than 80% below 40 mmHg and about 40% of patients had one reading of 30 mmHg or less. The fraction of time during the 24-hour on which patients remained with DPP below a given cut-off value is reported in Table 3. During the 24-hour curve, these patients were exposed to DPP < 50, 40, 30 mmHg for respectively 3 h 30’ ± 2 h 16’ (range, 30’; 10 h), 1 h 7’ ± 1 h 2’ (0; 5 h 30’), 10’ ± 16’ (0; 1 h 30’), being respectively 13.9 ± 9.0 (range, 1; 40), 4.7 ± 4.1 (0; 22), 1.0 ± 1.2 (0; 6) the number of time-points at which DPP fell below the given value. No statistically significant difference was found between the two study treatments. Thirty-eight percent of low DPP values were found during office hours (8 am-4 pm), compared to 62% outside office-hours. Twenty-nine percent of low DPP occurred at night.

**Table 3:** **Exposure time (during a 24-hour period) to DPP below given cut-off values**

DPP cut-off	Mean (hours, minutes)	sd	Range
<50 mmHg	3 h 30’	2 h 16’	30’;10 h
<45 mmHg	2 h 6’	1 h 40’	0; 8h
<40 mmHg	1 h 7’	1 h 2’	0; 5 h 30’
<35 mmHg	26’	30’	0; 2 h
<30 mmHg	10’	16’	0; 1 h 30’

DPP: diastolic perfusion pressure; sd: standard deviation.

## Discussion

The results of this study show that both IOP and PP can be very similar in a group of patients treated with bimatoprost or with LTFC. In fact, with the exception of SBP and SPP that were higher in the group receiving bimatoprost, all other comparisons did not show any statistically significant difference. No major effect of timolol in lowering PP could be evidenced in this trial. As a secondary result, we found that low DPP values were a common finding in our trial population, despite this sample had a very well controlled 24-hour IOP. Nearly all cases had at least one DPP reading below 50 mmHg and 40% below 30 mmHg. Furthermore, on average, patients were exposed to a DPP <50 mmHg for more than 3 hours per day.

The effect of timolol on PP is debated. A recent 24-hour study found that timolol (both solution and gel formulations) had no relevant effect on BP and PP in patients with POAG [25]. In another clinical trial where the timolol add-on therapy in prostaglandin treated glaucoma patients was evaluated, larger PP fluctuations were recorded after timolol [26]. In another recent prospective cross-over study, timolol was found to cause retinal vascular dysregulation in response to posture change in 30% of the trial sample after 6 weeks of treatment [27]. In our trial, timolol did not seem to have a clinically relevant effect in influencing the 24-hour PP.

A secondary, yet important finding of the present trial, was the recording of low nocturnal DPP values in a group of glaucoma patients. The clinical relevance of such a finding is not known but it certainly deserves some discussion. The role of PP in the pathogenesis (and progression) of glaucoma is not completely clear, although large epidemiologic studies have shown that ocular PP is a risk factor for the prevalence, incidence and progression of glaucoma [28–32]. Data from clinical trials seem to confirm the importance of PP: in the Early Manifest Glaucoma Trial (EMGT) [3], patients with low SPP tended to progress faster, and low SSP was a significant predictor of progression with an almost 50% higher risk; a recent report from the “Low-pressure Glaucoma Treatment study” showed that lower mean ocular perfusion pressure increased the risk for reaching a progression outcome [11].

A high proportion of patients in this trial was exposed to low PP values for a variable amount of time. It is hard to tell whether this risk is clinically relevant; unfortunately, the study was not designed with this objective and the short-term assessment of IOP and PP, the type of patients included (young and not selected according to the risk for low PP) are strongly limiting the possibility of a correct interpretation of our findings. Only long-term clinical trials assessing both visual field progression and 24-hour circadian IOP and PP in high-risk (e.g. older patients, patients treated with evening-dosed systemic anti-hypertensive medications) glaucoma patients will provide a conclusive answer. Other study limitations include: 1. Holter pressures and IOPs were not recorded at the same time; thus, at least potentially, all or some of the PP values that were derived from Holter could be wrong. On the other hand, with such study design Holter readings were not influenced by sudden awakenings that could not be avoided when IOP was measured at night and may result in a better estimate of the “true” pressures occurring during the sleeping period; 2. Patients in the trial were part of a highly selected population: they had well controlled IOP values, and, belonging to a study group, they probably tended to better adhere to treatment protocol; as a results they were likely to have better IOPs and PPs than the general glaucoma population. This, of course, decreases the generalizability of the findings; 3. The timing of drugs administration that could have influenced our findings: in this trial LTFC was administered in the morning (as suggested when the study was planned) while there is now evidence that it can be more effective when given in the evening [33, 34]. The strengths of this study should be also reported. First, at least to our knowledge, this is the biggest randomized clinical trial ever carried out with 24-hour circadian IOP and PP as outcomes in glaucoma patients. Second, the multicentre design and the stability of the IOP profile under the 2 study drugs reinforce the validity of our observations. The vast majority of the evidence about the role of PP in glaucoma comes from large epidemiologic studies or clinical trials where information on IOP is limited to single or few observations. It is reasonable to assume that our study could provide a more accurate estimate of the PPs in a POAG and OH patient population.

The “true” role of IOP variability and, consequently, PP in the pathogenesis of POAG and its progression will remain debated until a reliable tool to assess continuous IOP (and PP) will be clinically available. It seems we are not there yet; surrogate methods, as the one we used in this trial are probably the best options to get an estimate of short-term IOP variability and PP, though, we are aware, still far from perfection.

## Conclusions

In conclusion, low values of DPP are a common findings in glaucoma patients, even when IOP is under optimal control. A significant proportion of such patient can be exposed to “risky” PP values for a certain amount of time. The clinical meaning of this finding is still to be determined.
